# Iterative sorting reveals CD133+ and CD133- melanoma cells as phenotypically distinct populations

**DOI:** 10.1186/s12885-016-2759-2

**Published:** 2016-09-09

**Authors:** Carole Grasso, Matthew Anaka, Oliver Hofmann, Ramakrishna Sompallae, Kate Broadley, Winston Hide, Michael V. Berridge, Jonathan Cebon, Andreas Behren, Melanie J. McConnell

**Affiliations:** 1 Malaghan Institute of Medical Research, P.O. Box 7060, Wellington, 6242 New Zealand; 2 Ludwig Institute for Cancer Research, Olivia Newton-John Cancer & Wellness Centre, Austin Hospital, Heidelberg, VIC 3084 Australia; 3 Harvard T.H. Chan School of Public Health, 677 Huntington Avenue, Boston, MA 02115 USA; 4 Harvard Stem Cell Institute, Holyoke Center, Suite 727W, 1350 Massachusetts Avenue, Cambridge, MA 02138 USA; 5 Sheffield Institute for Translational Neuroscience, The University of Sheffield, 385a Glossop Road, Sheffield, S10 2HQ UK

## Abstract

**Background:**

The heterogeneity and tumourigenicity of metastatic melanoma is attributed to a cancer stem cell model, with CD133 considered to be a cancer stem cell marker in melanoma as well as other tumours, but its role has remained controversial.

**Methods:**

We iteratively sorted CD133+ and CD133- cells from 3 metastatic melanoma cell lines, and observed tumourigenicity and phenotypic characteristics over 7 generations of serial xeno-transplantation in NOD/SCID mice.

**Results:**

We demonstrate that iterative sorting is required to make highly pure populations of CD133+ and CD133- cells from metastatic melanoma, and that these two populations have distinct characteristics not related to the cancer stem cell phenotype. In vitro, gene set enrichment analysis indicated CD133+ cells were related to a proliferative phenotype, whereas CD133- cells were of an invasive phenotype. However, in vivo, serial transplantation of CD133+ and CD133- tumours over 7 generations showed that both populations were equally able to initiate and propagate tumours. Despite this, both populations remained phenotypically distinct, with CD133- cells only able to express CD133 in vivo and not in vitro. Loss of CD133 from the surface of a CD133+ cell was observed in vitro and in vivo, however CD133- cells derived from CD133+ retained the CD133+ phenotype, even in the presence of signals from the tumour microenvironment.

**Conclusion:**

We show for the first time the necessity of iterative sorting to isolate pure marker-positive and marker-negative populations for comparative studies, and present evidence that despite CD133+ and CD133- cells being equally tumourigenic, they display distinct phenotypic differences, suggesting CD133 may define a distinct lineage in melanoma.

**Electronic supplementary material:**

The online version of this article (doi:10.1186/s12885-016-2759-2) contains supplementary material, which is available to authorized users.

## Background

The heterogeneity and tumourigenicity of metastatic melanoma has been widely debated. Originally attributed to a stochastic model of clonal evolution [1], in recent years it has been proposed to follow a cancer stem cell model [2–6]. This model suggests tumour initiation, growth and recurrence is driven by a sub-population of tumourigenic cells that undergo stem cell-like asymmetric division to self-renew and produce hierarchical lineages of phenotypically differentiated, non-tumourigenic cells. However, the evidence that melanoma follows a cancer stem cell model is disputed [7–10]. Variations in methodology, from the reliability of xenografting melanoma cells taken directly from the patient, to how immuno-compromised mice need to be to accurately assess tumourigenicity, have raised doubts of the validity of a cancer stem cell model for melanoma [11, 12].

Key evidence supporting a melanoma cancer stem cell model has come from isolating cells that differentially express stem and progenitor cell markers, or chemo-resistance markers, and comparing their tumourigenic ability. In the case of melanoma, cells expressing the surface markers CD133 [4, 13] and ABCG2 [4], ABCB5 [14] and CD271 [15–17] have been examined, as well as the intracellular enzyme Aldehyde Dehydrogenase [18]. These studies claim there is a distinct lineage of melanoma stem cells, with marker-positive cells having greater tumourigenicity than marker-negative cells, and that only marker-positive cells have the ability to recapitulate the phenotypic heterogeneity of parental tumours [14].

In contrast, a study of 22 heterogeneously expressed markers from stage II, III and IV patient melanomas, including CD271, ABCB5, [7] and CD133 [8] reported that all cells, whether marker-positive or -negative, had tumourigenic capacity when assayed in highly immune-deficient hosts. In addition, tumours derived from both –positive and -negative cells recapitulated the complete spectrum of marker expression observed in the original tumour. These data implied that surface marker expression is reversible and does not mark any particular lineage. Instead, phenotype switching occurs in melanoma, with tumourigenicity driven by microenvironment switches from a proliferative to an invasive phenotype [19–22]. Other studies examining lineage and tumourigenicity have been similarly conflicted. Roesch et al. defined a slow-cycling lineage of JARID1B-positive cells as essential for continuous tumour growth [6], whereas Held et al. demonstrated multiple distinct populations with varying tumourigenic ability after single-cell engraftment of CD34 and CD271 subsets [17].

To investigate the relationship between cancer stem cells, tumourigenicity and surface marker expression, we studied the cell surface marker CD133 in primary melanoma cell lines. CD133 has been shown to be in part co-expressed with ABCB5 and CD271 [23–27], and has been used as a stem cell and cancer stem cell marker in melanoma [4, 28, 29], glioblastoma [30], colorectal cancer [31, 32] and others. While stressors such as hypoxia, chemotherapy and metabolic defects induce CD133 expression, the role in tumourigenesis is still not understood.

CD133+ and CD133- cells were sorted from 3 primary melanoma cell lines, and tumourigenicity and phenotypic characteristics observed over 7 generations of serial xeno-transplantation in NOD/SCID mice. We show for the first time the necessity of iterative sorting to isolate pure marker-positive and marker-negative populations for comparative studies of marker-positive cells in tumours, and present evidence that despite CD133+ and CD133- cells being equally tumourigenic, CD133 defines two phenotypically distinct populations in metastatic melanoma.

## Methods

### Cells and cell Culture

**Fig. 1:**
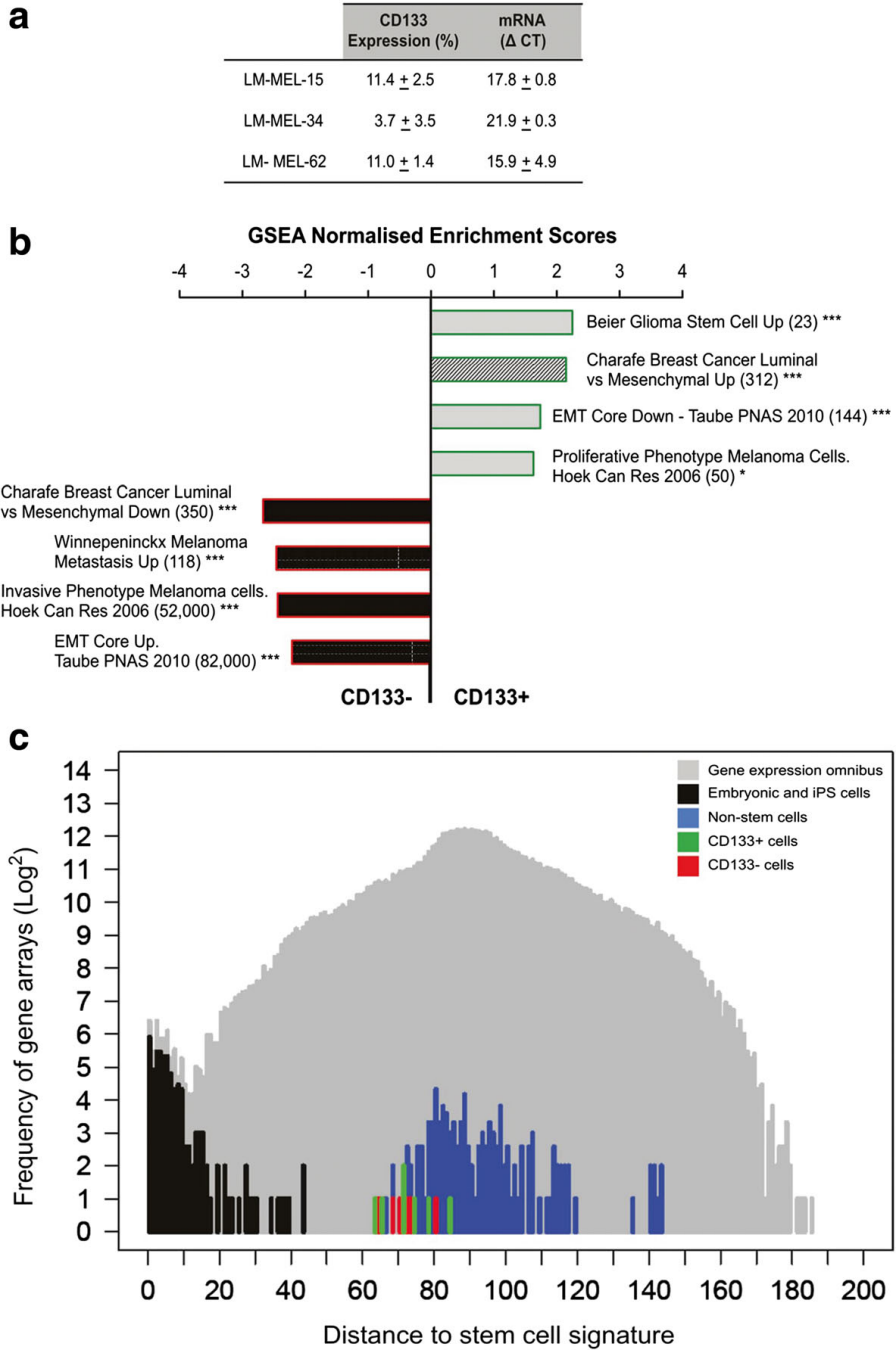
CD133+ and CD133- are not stem-like but display distinct phenotypes. **a** CD133 cell surface expression quantified by flow cytometry in 3 human primary lines derived from Stage IV melanoma metastases (LM-MEL-15, LM-MEL-34, LM-MEL-62). CD133 mRNA expression measured by real-time RT-PCR. Average of 3 replicate experiments +/- standard error. **b** Gene-expression profile for CD133+ (*right bars*, *green*) and CD133- (*left bars*, *red*) from 7 human primary malignant melanoma lines, using Gene Set Enrichment Analyses (GSEA) with a 5 % False Discovery Rate cut-off. Selected gene sets shown, with gene set size given in parentheses. Shading shows percentage of genes contributing to Enrichment Score: hatching, 20–50 %; grey, 50–75 %; black, 75–100 %. Nominal P values: *, *P* < 0.05, **, *P* <0.01, ***, *P* < 0.001. **c** Distribution pattern of CD133+ (*green*) and CD133- (*red*) microarray gene expression profiles compared to human Embryonic Stem (hES), induced Pluripotency Stem (iPS) cells (*black*), and fibroblasts and melanocytes (*blue*). Grey depicts global distribution of gene arrays in the Gene Expression Omnibus (GEO) database

This study utilized seven human melanoma cells lines (<15 passage) previously established from Stage IV malignant melanoma [33]. Ethical approval to use these cell lines for research purposes has been granted by the Austin Health Human Research Ethics Committee. All 7 lines were used in the GEO and GSEA studies (Fig. 1b and c), with LM-MEL-15, LM-MEL-34, and LM-MEL-62 (established from metastatic lymph node axilla), used in all other experiments, unless otherwise stated. LM-MEL cell lines were cultured in RPMI-1640 medium supplemented with 10 % fetal bovine serum (Sigma-Aldrich, Auckland, NZ), 100 units/mL penicillin, 100 μg/mL streptomycin, 0.1 % mercaptoethanol, 2 mM glutamax, and 25 mM HEPES. All plasticware was obtained from Thermo Scientific, NZ and all cell culture reagents from Invitrogen (Auckland, New Zealand).

### Magnetic-Activated Cell Sorting (MACS) for GEO and GSEA Studies

Cells were separated into CD133+ and CD133- populations using magnetic beads according to the manufacturers protocol (Miltenyi Biotec, Bergisch Gladbach, Germany) as described previously [34]. Briefly, LS columns were used for positive selection followed by depletion with LD columns. Columns were run in serial to enhance purity. Sorting results were tested for purity by flow cytometry.

### Gene Expression Omnibus (GEO)

RNA from CD133+ and CD133- cells was hybridized to Affymetrix U133A gene chips following standard Affymetrix protocols at the Memorial Sloan-Kettering Cancer Center Genomics Core Laboratory. For determination of stem cell signatures, gene expression data was downloaded from the National Center for Biotechnology Information GEO database and each sample rank-transformed. The expression of each pathway was scored by a modified, one-sided Kolmogorov–Smirnov statistic - based on the GSEA algorithm applied to single samples. The transformation was such that the null distribution, N(0,1), was determined from the cumulative distribution function of the background distribution score (10,000 gene name permutations). For each pathway the resulting normalized score was standardized against all samples of the same platform found in GEO using the ternary representation -1 and +1 for the top and bottom, 15 % and 0 otherwise.

### Gene Set Enrichment Analysis (GSEA)

RNA samples from cells sorted based on CD133 expression using MACS were analyzed on Affymetrix HG-U133A microarrays at the Memorial Sloan-Kettering Cancer Centre Genomics Core Laboratory. Raw data were normalized using the Robust Multiarray Average (RMA) method [35]. Gene Set Enrichment Analysis [36] was performed using the RMA normalized data to calculate the Enrichment Score (ES), with gene set permutation and a 5 % False Discovery Rate cutoff. A nominal *p* value was used to estimate the statistical significance of the ES (*, *P* < 0.05, **, *P* <0.01, ***, *P* < 0.001.). Gene sets included those curated in category C2 of the MSigDB database v3.0, as well as additional melanoma and cancer-related gene lists derived from recent publications. Full GSEA data available in Supporting Information.

### Flow Cytometry and Fluorescence-Activated Cell Sorting (FACs) for xenotransplantation

Cells, suspended in HBSS at 2 × 10^6^ cells/mL, were incubated in the dark for 30 mins at room temperature with 1:100 dilution of AC133, 293C, or the appropriate mouse IgG1 or mouse IgG2b isotype control antibodies, all directly conjugated to R-PE (Miltenyi Biotech, Germany). Cells were washed then analyzed on a BD FACSort (Becton Dickson, CA) using propidium iodide as a viability dye. CD133 expression on viable cells was analyzed with FlowJo software (TreeStar, CA). For sorting experiments, cells were sorted at 20 psi sheath pressure, with a 100 μm nozzle. Sort gates were set at the top and bottom 5 % of viable CD133-PE histogram.

### Mice

Breeding pairs of the inbred strain NOD/SCID were obtained from the Hercus-Taieri Research Unit, University of Otago, New Zealand. All mice were maintained at the Biomedical Research Unit of the Malaghan Institute of Medical Research. Experimental protocols were approved by Victoria University Animal Ethics Committee, Wellington, New Zealand, and performed according to institutional guidelines. Mice were 6 to 12 weeks of age.

### Tumour preparation and serial transplant

2 × 10^5^ cells were suspended in 50 μL of Geltrex (Invitrogen, Auckland NZ) and injected sub-cutaneously into NOD/SCID mice, 3–5 mice per group. Tumour growth was recorded at 3–6 days, and when ~200 mm^3^, mice were culled by cervical dislocation and tumours excised. Tumours from each animal in the group were pooled, mechanically dissociated and filtered through 100, 70 and 40 μm filters into a single cell suspension, then plated into tissue culture plates in 20 mL of cell culture media. The following day, media was replaced, leaving adherent melanoma cells. Cultures were expanded by passage as required. Cells were lifted with 0.05 % Trypsin-EDTA (Life Technologies, NZ), and washed 3 times before being resuspended in Geltrex for the next injection.

### Statistical analysis

Basic statistical analyses of tumour growth and CD133+ cell number (mean and standard error) were carried out using the appropriate packages in Excel (Microsoft) or Prism (Graphpad). More complex statistical analyses of cell surface CD133+ cell numbers or CD133 transcript could not be performed due to limited size of datasets. Statistical significance of GSEA data was determined using standard parameters [36].

### RNA extraction and Real Time-PCR

RNA from freshly excised pooled tumours, or from cells in culture, was extracted using a RNeasy kit (QIAGEN, Valenica, CA). cDNA was synthesized using I-Script c-DNA Synthesis (BioRad, Auckland, NZ). Real-time PCR was performed using primers to 18 s (QT00199367) and CD133 (QT00075586), with QuantiTect SYBR Green PCR Master Mix (QIAGEN, Valenica, CA) in the 7500 Real Time PCR System (Applied Biosystems, Carlsbad, CA), according to manufacturer’s instructions. Cycle threshold (Ct) for CD133 was normalized to 18 s rRNA Ct (ΔCT). ΔCT was normalized between the vehicle and treated cell RNA (ΔΔCt). Amplification efficiency of all primers were equivalent so fold change was determined by 2^-ΔΔCt^. The CD133 primers amplified exons 5, 6 and 7, and detected all known mRNA variants. Caco-2 cells were used as a positive control for CD133 expression.

### Immunohistochemistry

Following dewaxing, 4 mm paraffin sections of tumours were dehydrated in graded ethanol, endogenous peroxidase activity blocked for 10 min in 3 % hydrogen peroxide, and antigens retrieved by microwave boiling in 10 mM citrate buffer pH 6 (cat. no. TA-050-CBX, Thermo Fisher) followed by 20 min cooling. Sections were incubated for 45 min at room temperature in 1:700 mouse-anti-human-CD133 antibody (ahE2, mAb80B258, kindly provided by Dr Denis Corbeil, Max-Planck-Institute of Molecular Cell Biology and Genetics, Dresden, Germany). mIgG1 (X0931, DAKO) of matching concentration served as negative control. Binding was detected with anti-mouse-HRP polymer (cat. No. K4004, Envision™+, DAKO, 1 h, room temperature), and visualized with AEC. Nuclei were counterstained with Mayer’s Hemotoxylin (7 s). Slides were mounted with crystal mount and subsequently cover slipped with Pertex.

### Image analysis

IHC images were analysed using Aperio slide viewer (http://www.aperio.com).

## Results

### CD133+ and CD133- melanoma cells have distinct phenotypes

This study utilized human malignant melanoma cells lines (<15 passage) previously established from Stage IV melanoma [33]. Surface CD133 expression was determined by flow cytometry for 3 out of the 7 cell lines (LM-MEL-15, LM-MEL-34, and LM-MEL-62). Expression varied from an average of 3.7 % for LM-MEL-34, to ~11 % for LM-MEL-15 and -62 cells. Quantitative RT-PCR demonstrated that CD133 transcript correlated with surface CD133 protein, with LM-MEL-15 and -62 displaying similar levels, and LM-MEL-34 cells having the lowest proportion (Fig. 1a).

We asked whether there was a transcriptional profile specifically associated with CD133+ melanoma cells. A larger panel of 7 LM-MEL human primary malignant melanoma cell lines, including −15, −34 and −62 were sorted into CD133+ and CD133- populations by immuno-magnetic cell sorting (MACS), RNA harvested and the gene expression profile of CD133+ and CD133- queried by microarray. Data were analyzed via Gene Set Enrichment Analysis [36], (Fig. 1b, see Additional file 1: Table S1 for full list). CD133+ cells could be segregated from CD133- cells by several pathways. In particular, CD133+ cells resembled both glioma stem cells and highly proliferative melanoma cells, and had low expression of genes associated with a mesenchymal phenotype, including the epithelial to mesenchymal transition of breast cancer. CD133- cells had gene expression resembling invasive, metastatic melanoma, with increased expression of the epithelial to mesenchymal transition genes. These data indicated clear differences between CD133+ and CD133- cells.

There are many reports of CD133 as a cancer stem cell marker, and the GSEA data suggested that CD133+ cells resembled glioma stem cells. One of the key characteristics of cancer stem cells is self-renewal and asymmetric division mediated by expression of embryonic stem cell genes. To determine whether the LM-MEL CD133+ cells had preferential expression of stem cell genes, expression of CD133+ and CD133- cells from the 7 LM-MEL cell lines were compared to gene expression signatures from ~300 human embryonic stem (hES), and inducible pluripotent stem cells (iPS) taken from a Gene Expression Omnibus (GEO). hES and iPS are known to express CD133 [37]. As a comparison, these profiles were analyzed against gene expression of fibroblasts and melanocytes and all microarray data in the GEO database (grey), (Fig. 1c). As would be expected, hES and iPS gene expression profiles were the most stem-like, falling at the far left of the spectrum (black). Expression profiles from fibroblasts and melanocytes had much lower representation of the stem-like metric (blue), consistent with their differentiated state. The gene expression of CD133+ (green) and CD133- (red) cells was more stem-like than the differentiated melanocytes, consistent with the increased self-renewal activity and stem cell gene expression observed in cancer cells. However, the equal distance of CD133+ and CD133- cells from the hES/iPS stem cell signature implied that there was no differential stem cell gene expression between CD133+ and CD133- cells.

### Effective depletion of CD133-expressing cells requires iterative sorting

**Fig. 2:**
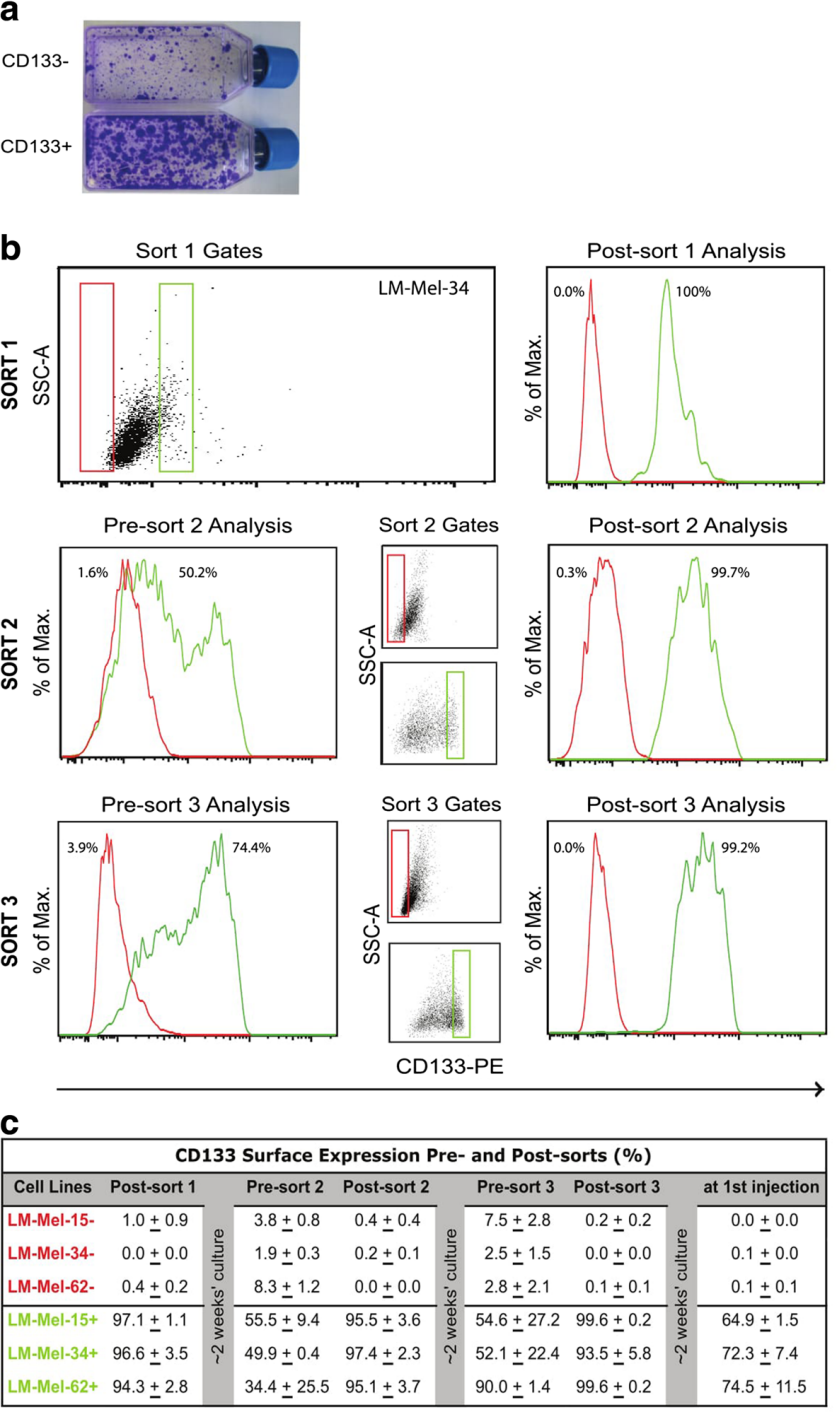
Serial cell sorting is essential to isolate highly pure CD133+ and CD133- populations. **a** Cell proliferation after seeding 10^5^ LM-MEL-62 CD133- and CD133+ cells, from an initial FACS sort. Cells stained with crystal-violet after two weeks’ growth. **b** Serial FACS cell sorting example of CD133+ (*green*), and CD133- (*red*) melanoma cells from primary cell line LM-MEL-34. **Sort 1**: Sort gates set to collect extreme 5 % of population (Sort 1 Gates), with resulting sorted populations shown (Post-sort 1 Analysis). **Sort 2**: Sort 1 cells cultured for ~2 weeks and CD133 expression re-analyzed (Pre-sort 2 Analysis). Cells re-sorted (Post-sort 2 Analysis). **Sort 3**: Sort 2 cells cultured and CD133 re-analyzed (Pre-sort 3 Analysis), then re-sorted (Post-sort 3 Analysis). Data representative of 2 replicate experiments per cell line. **c** Percentage CD133 surface expression by flow cytometry of CD133+ and CD133- cells from 3 primary cell lines pre- and post- 3 sorts and on day of injection. Data are representative of 2 replicate experiments +/- standard error

To identify whether the differential gene expression identified by GSEA impacted on tumourigenicity of CD133- and CD133+ cells, LM-MEL-15, -34, and -62 were sorted into CD133+ and CD133- populations by fluorescent-activated cell sorting (FACS). FACs sorting was chosen at this stage, to ensure optimum purity of CD133+ and CD133- cells for tumourigenic studies. Initial sorting of LM-MEL-62 cells into CD133+ and CD133- cells and subsequent culture demonstrated a difference in growth rate in vitro (Fig. 2a), consistent with the proliferative signature identified by GSEA.

To accurately sort CD133+ and CD133- cells, only the highest and lowest 5 % of CD133-stained cells were rigorously sorted by FACS from LM-MEL-15, -34, and -62. The initial sort (Sort 1) purified CD133+ cells to a population >94.3 % CD133+, and CD133- to greater than 99 % purity, (Fig. 2b, Sort 1 representative, Fig. 2c, Post-sort 1). After several days in culture to expand the viable population, surface CD133 was re-measured (Fig. 2c, Pre-sort 2). Cells with cell surface CD133 dropped to 34.4–55.5 % in the CD133+ population, but more notably CD133 expression increased in the CD133- population, to 1.9–8.3 %. This emergence of CD133+ cells in the CD133- population suggested the possibility of transient loss of CD133 from the surface of a CD133+ cell at the time of sorting.

To improve purity, sorting of both populations was repeated (Fig. 2b, Sort 2 representative), with CD133+ cells re-purified to >95.1 % of cells expressing CD133, and CD133- with <0.4 % (Fig. 2c, Post-sort 2). Again, cells were left to recover, then CD133 surface expression re-measured. A greater proportion of CD133+ cells retained cell surface CD133 - 52.1–90.0 % (Fig 2c, Pre-sort 3), while again CD133+ cells in the CD133- population increased to 2.5–7.8 %.

A third sort (Fig. 2b, Sort 3 representative) was then performed to deplete these CD133+ cells from the CD133- population (Fig. 2c, Post-sort 3). These finally remained stably depleted of CD133 for 2 weeks in culture (<0.1 %), (Fig. 2c at 1st injection). As seen previously, CD133+ cells were purified to >93.5 %, but reverted back to a mixed population, with 25–35 % of CD133+ cells no longer displaying CD133 on the cell surface. This iterative sorting procedure was used to produce highly depleted CD133- cells, and enriched CD133+ cells, for each experiment.

### CD133+ and CD133- cells were equally tumourigenic

We next looked at 2 characteristics often associated with cancer stem cells in vivo – tumour initiation and tumour propagation. Highly purified CD133+ and CD133- cells from LM-MEL-15, -34 and -62, with greater than 95 % viability, were injected sub-cutaneously into NOD/SCID mice and allowed to form tumours. Titration of the starting inoculum of 100,000, 10,000 and 1,000 CD133+ and CD133- cells did not alter tumour initiation, even at the lowest cell number (Additional file 2: Figure S1). This suggested there was no loss of tumour-initiating cells with depletion of CD133.

**Fig. 3:**
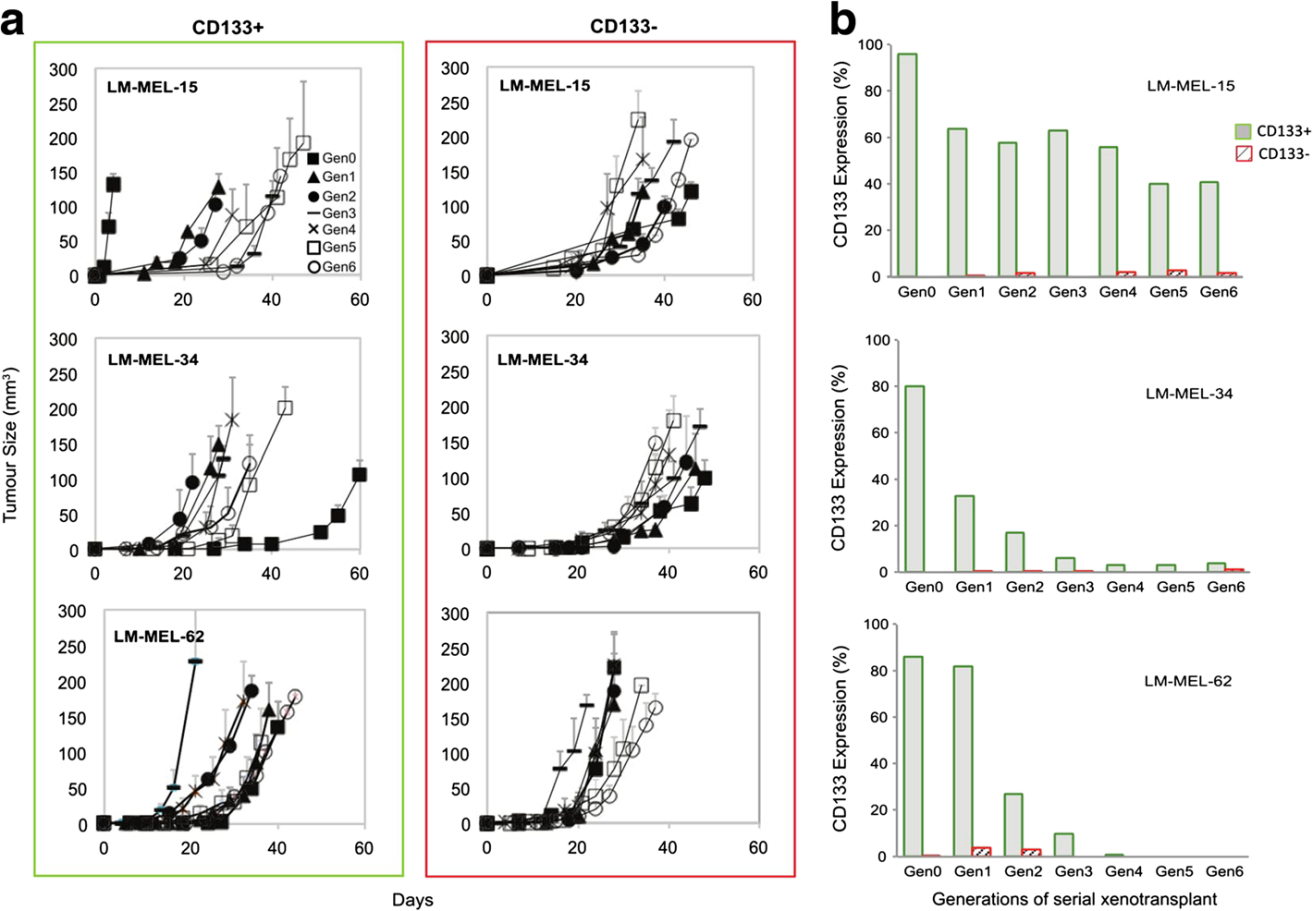
CD133+ and CD133- cells are equally tumourigenic with distinct phenotypes. **a** Serial xeno-transplantation of CD133+ and CD133- cells over 7 generations (Gen0 to Gen6) in NOD/SCID mice. Serially sorted cells from 3 primary cell lines were injected sub-cutaneously (3–5 mice per group) and tumours grown to ~200 mm^3^. Tumours were pooled, resuspended and cultured for ~2 weeks before reinjection. Data representative of 2 replicate experiments. **b** Flow cytometry of CD133 surface expression of CD133+ and CD133- cells prior to injection into NOD/SCID mice, over 7 generations (Gen0–Gen6). Data representative of 2 replicate experiments

A serial xeno-transplantation experiment was undertaken to determine the ability of CD133+ and CD133- cells to maintain on-going tumour growth. For each LM-MEL cell line −15, −34 and −62, sub-cutaneous tumours were established in 3 to 5 NOD/SCID mice, using 10^5^ highly purified, >95 % viable, early passage CD133+ or CD133- cells. Tumours were allowed to grow to ~200 mm^3^ (Generation 0, or Gen0), then excised, pooled and a single cell suspension plated to select the adherent melanoma cells from the host stromal component. These cells were cultured for ~2 weeks to ensure stability and purity, before surface CD133 expression was quantified and cells re-injected into mice (Gen1). No further sorting of CD133+ and CD133- cells occurred after Gen0 in order to analyze the dynamic characteristics of the original highly-purified populations. Both CD133+ and CD133- cells from each LM-MEL line initiated tumour formation and growth over 7 generations (Gen0 to Gen6), (Fig. 3a). The iterative sorting and serial transplantation experiment was repeated, and in both independent replicate experiments, depletion of the CD133+ population did not alter tumour formation. This demonstrated that there was no loss of tumour propagation activity with CD133 depletion. Together with the retention of tumour initiation, these data demonstrated there was no cancer stem cell activity specifically associated with CD133+ cells, consistent with the hES gene signature analysis.

Differences in proliferation rate between CD133+ and CD133- cells were suggested by the gene expression data, and observed in vitro, so we analysed growth of CD133+ and CD133- in vivo, using all the generations of transplanted tumours from all 3 LM-MEL cell lines.

We analyzed time-to-appearance of palpable tumours for each sorted population, and while there was a hint that CD133- tumours appeared more rapidly than CD133+, this difference was not statistically significant (data not shown). We also assessed whether there was a difference in growth rate between CD133- and CD133+ tumours by analyzing the area under the growth curve. There was a suggestion that CD133- tumours grew more quickly, but again this was not statistically significant (data not shown). However, there was a difference in in vitro cell surface expression of CD133+ and CD133- cells during serial transplant experiments. Tumour cells were cultured after each generation and CD133 surface expression measured prior to reinjection. CD133 expression on CD133- cells and CD133+ cells were phenotypically distinct, with CD133- expressing virtually zero CD133 throughout the 7 generations, as opposed to CD133+ cells that retained high expression in LM-MEL-15 cells, with tumours derived from LM-MEL-34 and -62 CD133+ cells showing gradual loss of cell surface CD133 over time (data representative of 2 replicate experiments, Fig. 3b).

#### CD133 could be induced in CD133- tumours in vivo

**Fig. 4:**
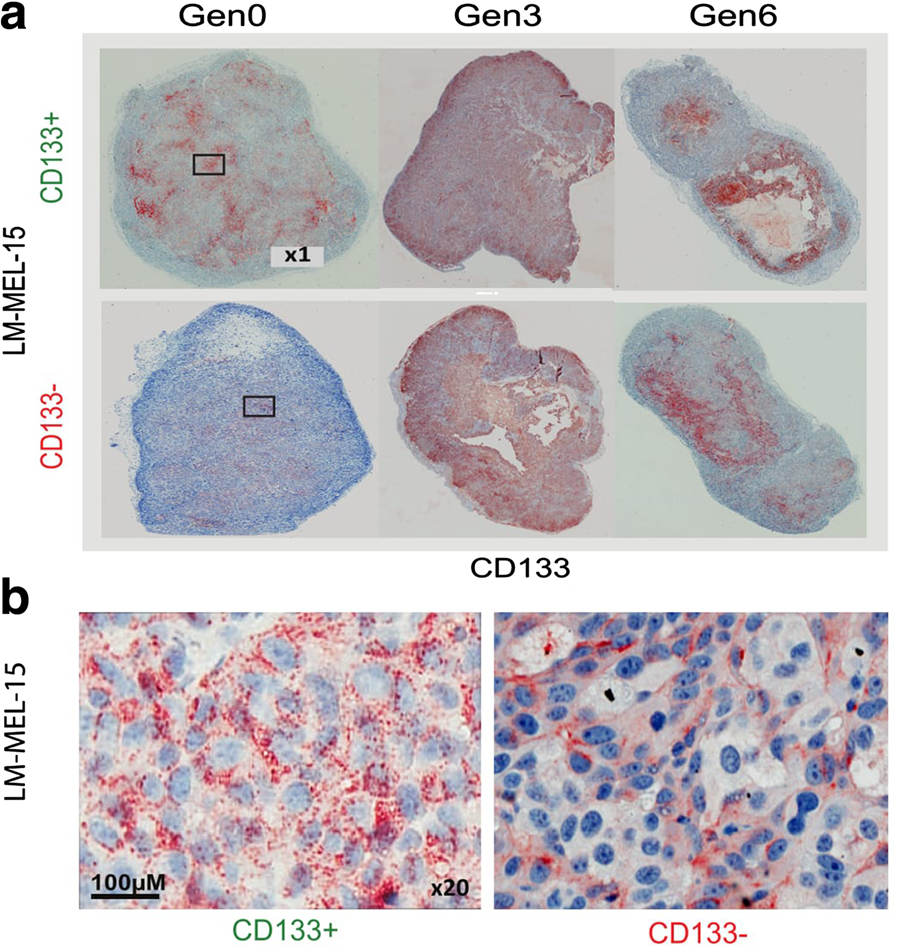
CD133- derived tumours express CD133 in vivo but cannot retain it in vitro. **a** Subcutaneous tumours derived from highly purified LM-MEL-15 CD133+ (*top*) and CD133- (*bottom*) were formaldehyde-fixed, paraffin-embedded and stained with CD133 antibody (ahE2). Images obtained at 1× magnification (Aperio slide viewer). **b** LM-MEL-15, Gen0 CD133 staining (20× magnification) for CD133+ (*left*) and CD133- (*right*) tumours

Due to the phenotypically distinct in vitro CD133 surface expression on CD133- and CD133+ cells between serial xenotransplantations, we assessed whether these differences were also evident in vivo. Tumour sections were stained with CD133 antibody (ahE2) at Generation 0, 3 and 6. Expression of CD133 was observed in most tumours throughout the serial xenotransplants (Fig. 4a and b). There was particularly high expression bordering necrotic tissue in some tumours, consistent with data describing a role for CD133 in hypoxia and other stress responses [38, 39]. Notably, both CD133+ and CD133- tumours expressed both cell surface and intra-cellular CD133 in vivo.

#### CD133- cells derived from CD133+ cells have a CD133+ phenotype

With equal CD133 expression in CD133+ and CD133- cells in vivo (Fig. 4a and b), yet distinct differences in gene expression profile (Fig. 1b), and CD133 expression in vitro (Fig. 3b), we asked whether CD133- cells derived from CD133+ cells were CD133+ cells that had transiently lost CD133 from the cell surface or whether they were a phenotypically distinct population from CD133 + .

**Fig. 5:**
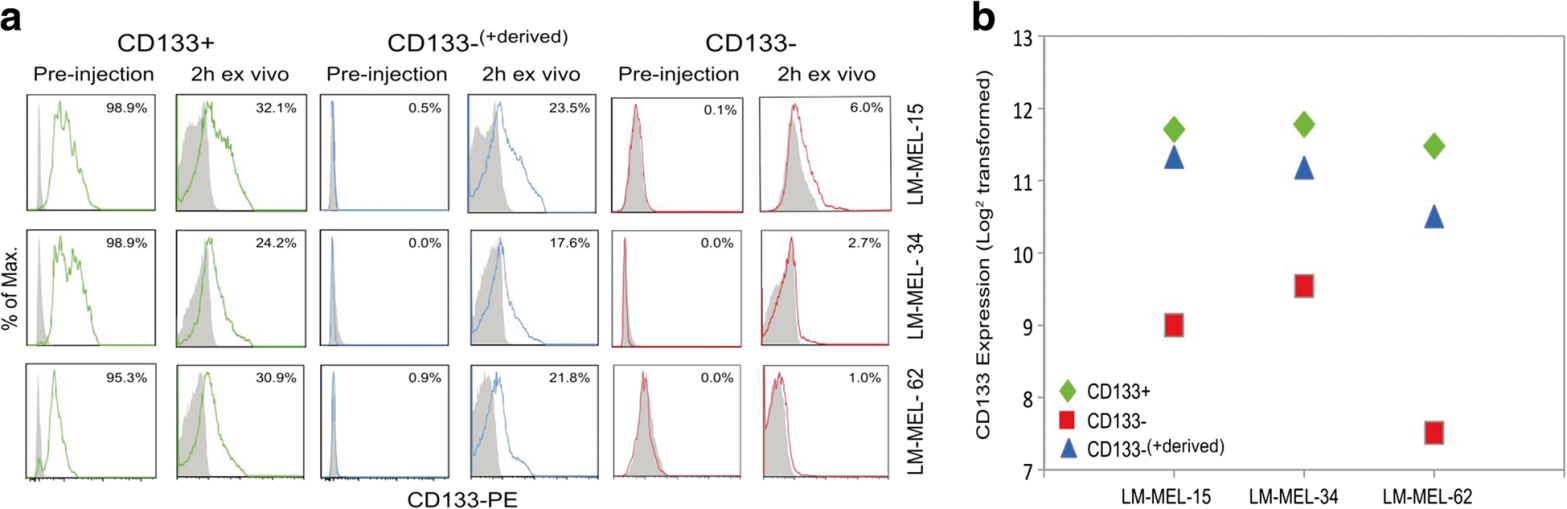
CD133- cells derived from CD133+ cells have a CD133+ phenotype. **a** Sort 3 CD133+ (*green*), and CD133- (*red*) cells were re-sorted and CD133- cells from the CD133+ population collected (CD133-^(+derived)^, *blue*). Sub-cutaneous tumours were established from these 3 populations (2 mice per group) and excised at ~200 mm^3^. Flow cytometry analysis of CD133 surface expression was performed on viable cells at 2 h post-excision. Signal from isotype control antibody, grey. **b** CD133+ (*green*), CD133- (*red*) and CD133-^(+derived)^ (*blue*) tumours were collected and CD133 transcript in each tumour quantified by microarray analysis. Data representative of 2 replicate experiments

LM-MEL-15, -34, -62 CD133+ cells post-Sort 3 were cultured for several days and cells that lost CD133 expression purified by FACS. These CD133- cells derived from CD133+ cells (named CD133-^(+derived)^) were then xenografted, as were CD133+ and CD133- cells from Sort 3. Once tumours reached 200 mm^3^, they were harvested and CD133 surface expression measured by flow cytometry immediately *ex vivo* (Fig. 4a). In all LM-MEL lines, CD133 expression in the CD133-^(+derived)^ tumours closely reflected the CD133+ tumours and not the CD133- tumours, suggesting derivation from, and retention of a CD133+ phenotype. The CD133 transcript in tumours was quantified by microarray analysis. The CD133-^(+derived)^ tumours appeared to have consistently higher CD133+ expression than the CD133- derived tumours (Fig. 5b). This demonstrated that CD133+ cells did not give rise to CD133- cells, but merely down-regulated CD133 from the cell surface.

Cell surface expression of CD133 in the CD133- tumours was not retained in vitro, with CD133 expression dropping after 24 h in culture (data not shown). This was unlike the retention of surface CD133 on CD133+ cells in vitro over the same time. This strengthened the hypothesis that highly purified CD133+ cells were a population phenotypically distinct from the CD133- cells, despite the levels of CD133 induced in these cells by the tumour microenvironment.

## Discussion

Following transcriptome analysis of CD133+ and CD133- cells from a panel of low-passage, primary tumour-derived melanoma cells, we established that each population had a distinct gene expression profile in vitro that was conserved across multiple primary melanoma cell lines. Serial xenograft experiments with 3 different metastatic melanoma cell lines demonstrated that despite their different gene profiles, highly purified CD133+ and CD133- populations were equally tumourigenic with similar proliferation rates.

The equivalent tumourigenicity of these melanoma subsets supports an earlier study that showed both CD133+ and CD133- populations to be equally tumourigenic [8], but contradicts findings from the same study that resulting tumours from both populations contained both CD133+ and CD133- cells with the same frequency as the parental tumour. One reason for this discrepancy may lie in the technical details of isolation of CD133+ and CD133- populations. We have shown that the initial CD133- population contained cells that were in fact CD133+, but had transiently lost CD133 from the surface at the time of sorting. When isolated, these CD133-^(+derived)^ cells induced CD133 in vivo exactly the same as the CD133+ cells, and had the same gene expression patterns as CD133+ cells, suggesting there are stable lineages of CD133+ cells in melanoma that are not interchangeable with CD133- cells.

The impact of this finding was that several rounds of sorting were essential for isolation of 99.9 % pure and in-vitro stable CD133- populations. We suggest that insufficient cell sorting may be partly responsible for the contradictory evidence of heterogeneity and lineage reported in melanoma stem cell experiments.

The frequency and equivalent representation of embryonic stem cell gene expression, and equivalent tumourigenicity of CD133+ and CD133- cells, demonstrated that in our cells CD133 did not mark a melanoma stem cell. Consistent with other studies, the actual frequency of the melanoma-initiating cell was high in each population, at least 1:1000 and probably higher. NOD/SCID mice were used throughout our study where engraftment is reduced compared to NOD/SCID/IL2Rg knock-out (NSG) mice. Transplantation into NSG mice, which have extreme defects in both adaptive and innate immunity, indicate that as many as 1:4 human melanoma cells may be capable of engrafting and initiating tumours [8] in this particular environment. We have not attempted to accurately quantify the tumour-initiating fraction in each CD133+ and CD133- population, but saw no overt difference. We also saw no difference in tumour growth. These data add to the growing body of evidence that the surface marker CD133 does not label a cancer stem cell/tumour initiating cell in melanoma, and clearly demonstrates that both CD133+ and CD133- populations contain cells capable of initiating and maintaining tumours. This is also evident in CD133+ cells in the brain tumour glioblastoma [40, 41], colorectal cancer [32, 42], lung cancer [43], as well as our data for melanoma.

Our cells were grown in fetal calf serum which could potentially mask differences in the cancer stem cell phenotype in CD133+ cells, however, growth of melanoma cells in stem cell media leads to the induction of a neural stem cell phenotype which is inappropriate for the analysis of melanoma cells [44]. In our study, there were clear differences observed in vitro between the CD133+ and CD133- lineages even in the presence of serum. In vitro, the CD133+ lineage remained functionally stable despite changes in expression induced by the microenvironment, as demonstrated by the *ex vivo* expression data. However, the proliferative differences suggested by GSEA and growth in vitro were obscured by the variability in CD133+ tumour cells, making none of the tumour growth data statistically significant.

The normal function of CD133, and its role in cancer is still essentially unknown. In glioblastoma, several differences have been observed between CD133- and CD133+ tumours, including proliferation, differentiation and angiogenesis [40, 45–48], with a recent report that the non-coding miR125b can block migration of CD133+, but not CD133- cells [49]. Knockdown of CD133 can delay tumour growth and CD133+ cells promote vasculogenic mimicry [26]. CD133 can be up-regulated in response to chemotherapy, hypoxia and other physiological stresses [38, 50–54]. We observed up-regulation of CD133 in vivo, in both CD133+ and CD133- melanoma cells. Interestingly we also observed up-regulation of intracellular CD133, which has recently been reported in several settings [55, 56]. Again, the function of intracellular CD133 is not known. While we have not attempted to identify the signals that led to CD133 up-regulation, the CD133- cells could not maintain that expression in vitro, further differentiating them from CD133+ cells. Whether this difference is only unmasked in the in vitro system as consistent culturing conditions may slow down or pause plasticity events, or if it is a consequence of it, remains to be clarified.

## Conclusions

We suggest there are distinct lineages in melanoma development, based on the phenotype of CD133+ and CD133- cells. CD133 expression and localisation is dynamic and driven in part by the cellular microenvironment, and the CD133+ lineage reported here does not exhibit continuous localization of CD133 to the cell surface. Rigorous purification was required to identify ‘true’ CD133- cells, since CD133+ cells can transiently downregulate CD133. This highlights the need to use functional assays, rather than simple marker expression for phenotypic, and phenotype switching, analysis [11]. Our data implies that at least 2 phenotypically distinct populations exist within metastatic melanoma - CD133+ and CD133-, as reported in glioblastoma [48]. Within the CD133+ and CD133- populations, environment-driven phenotype switching between initiation, proliferation and invasion can occur, but switching between CD133+ and CD133- lineages does not.

## Additional files

Additional file 1: Table S1. CD133+ and CD133- GSEA Raw Data. Full gene-expression profile of 7 primary human malignant melanoma CD133+ and CD133- cells using Gene Set Enrichment Analyses (GSEA) with a 5 % False Discovery Rate cut-off (PDF 277 kb) Additional file 2: Figure S1. CD133+ and CD133- cells have similar frequency of tumour-initiating cells. Serial dilution of CD133+ and CD133- cell used in sub-cutaneous xenograft. Square, 10^5^ cells; triangle, 10^4^ cells; circle, 10^3^ cells. Average (+/- SD) tumour volume measured over time, 3–5 mice/group. Data representative of 2 independent replicate experiments (PDF 203 kb)
