# Supplementary material for: Iterative sorting reveals CD133+ and CD133- melanoma cells as phenotypically distinct populations
Source: BMC Cancer. 2016 Sep 9;16(1):726. doi: 10.1186/s12885-016-2759-2 (PMC5017126; doi:10.1186/s12885-016-2759-2)
Supplement: Additional file 1: Table S1. — CD133+ and CD133- GSEA Raw Data. Full gene-expression profile of 7 primary human malignant melanoma CD133+ and CD133- cells using Gene Set Enrichment Analyses (GSEA) with a 5 % False Discovery Rate cut-off (PDF 277 kb) [file 12885_2016_2759_MOESM1_ESM.pdf]

TABLE S1.

Full list of Gene Set Enrichment Analysis for 7 LM-MEL CD133+ and CD133- cells

CD133+

Gene Set Name	NES	p-value	FDR
BEIER_GLIOMA_STEM_CELL_UP	2.25	0.000	0.030
ROY_WOUND_BLOOD_VESSEL_DN	2.21	0.000	0.037
GRAHAM_CML_DIVIDING_VS_NORMAL_DIVIDING_DN	2.18	0.000	0.047
ELLWOOD_MYC_TARGETS_DN	2.16	0.000	0.038
CHARAFE_BREAST_CANCER_LUMINAL_VS_MESENCHYMAL_UP	2.15	0.000	0.038
AIGNER_ZEB1_TARGETS	2.07	0.000	0.048
EMT_CORE_DOWN [TAUBE 2010 PNAS]	1.74	0.000	0.019
PROLIFERATIVE PHENOTYPE MELANOMA CELLS [HOEK 2006 PIG CELL MEL RES]	1.63	0.013	0.024

CD133-

Gene Set Name	NES	p-value	FDR
SOTIRIOU_BREAST_CANCER_GRADE_1_VS_3_UP	-2.86	0.000	0.000
KOBAYASHI_EGFR_SIGNALING_24HR_DN	-2.67	0.000	0.000
CHARAFE_BREAST_CANCER_LUMINAL_VS_MESENCHYMAL_DN	-2.67	0.000	0.000
SENGUPTA_NASOPHARYNGEAL_CARCINOMA_UP	-2.64	0.000	0.000
GRAHAM_NORMAL_QUIESCENT_VS_NORMAL_DIVIDING_DN	-2.62	0.000	0.000
GRAHAM_CML_DIVIDING_VS_NORMAL_QUIESCENT_UP	-2.62	0.000	0.000
FUJII_YBX1_TARGETS_DN	-2.6	0.000	0.000
RUIZ_TNC_TARGETS_DN	-2.6	0.000	0.000
RODRIGUES_THYROID_CARCINOMA_POORLY_DIFFERENTIATED_UP	-2.54	0.000	0.000
ROSTY_CERVICAL_CANCER_PROLIFERATION_CLUSTER	-2.52	0.000	0.000
CROONQUIST_IL6_DEPRIVATION_DN	-2.52	0.000	0.000
RODRIGUES_THYROID_CARCINOMA_ANAPLASTIC_UP	-2.51	0.000	0.000
BLUM_RESPONSE_TO_SALIRASIB_DN	-2.48	0.000	0.000
BASAKI_YBX1_TARGETS_UP	-2.47	0.000	0.000
KANG_DOXORUBICIN_RESISTANCE_UP	-2.46	0.000	0.000
WINNEPENNINCKX_MELANOMA_METASTASIS_UP	-2.46	0.000	0.000
INVASIVE PHENOTYPE MELANOMA CELLS [HOEK 2006 PIG CELL MEL RES]	-2.44	0.000	0.000
BERENJENO_TRANSFORMED_BY_RHOA_UP	-2.41	0.000	0.000
LE_EGR2_TARGETS_UP	-2.4	0.000	0.000
FERREIRA_EWINGS_SARCOMA_UNSTABLE_VS_STABLE_UP	-2.38	0.000	0.000
FOURNIER_ACINAR_DEVELOPMENT_LATE_2	-2.38	0.000	0.000
WHITEFORD_PEDIATRIC_CANCER_MARKERS	-2.36	0.000	0.000
PUJANA_BREAST_CANCER_WITH_BRCA1_MUTATED_UP	-2.34	0.000	0.000
REN_ALVEOLAR_RHABDOMYOSARCOMA_DN	-2.33	0.000	0.000
DODD_NASOPHARYNGEAL_CARCINOMA_DN	-2.31	0.000	0.000
CHARAFE_BREAST_CANCER_LUMINAL_VS_BASAL_DN	-2.31	0.000	0.000
MARKEY_RB1_ACUTE_LOF_DN	-2.3	0.000	0.000
PUJANA_XPRSS_INT_NETWORK	-2.3	0.000	0.000
CROONQUIST_NRAS_SIGNALING_DN	-2.27	0.000	0.000
JIANG_TIP30_TARGETS_UP	-2.27	0.000	0.000
SCHUETZ_BREAST_CANCER_DUCTAL_INVASIVE_UP	-2.26	0.000	0.000
RORIE_TARGETS_OF_EWSR1_FLI1_FUSION_UP	-2.26	0.000	0.000
STEIN_ESRRA_TARGETS_RESPONSIVE_TO_ESTROGEN_DN	-2.25	0.000	0.000
BROWNE_HCMV_INFECTION_24HR_DN	-2.25	0.000	0.000
NAKAMURA_TUMOR_ZONE_PERIPHERAL_VS_CENTRAL_UP	-2.25	0.000	0.000
BROWNE_HCMV_INFECTION_20HR_DN	-2.25	0.000	0.000
CLASPER_LYMPHATIC_VESSELS_DURING_METASTASIS_DN	-2.25	0.000	0.000
SHEDDEN_LUNG_CANCER_POOR_SURVIVAL_A6	-2.23	0.000	0.000
KIM_WT1_TARGETS_DN	-2.23	0.000	0.000
REN_BOUND_BY_E2F	-2.22	0.000	0.000
SONG_TARGETS_OF_IE86_CMV_PROTEIN	-2.22	0.000	0.000
EMT_CORE_UP [TAUBE 2010 PNAS]	-2.21	0.000	0.000
BRUECKNER_TARGETS_OF_MIRLET7A3_DN	-2.21	0.000	0.000
CROONQUIST_NRAS_VS_STROMAL_STIMULATION_DN	-2.21	0.000	0.000
ODONNELL_TFRC_TARGETS_DN	-2.21	0.000	0.000
MISSIAGLIA_REGULATED_BY_METHYLATION_DN	-2.21	0.000	0.000
VERNELL_RETINOBLASTOMA_PATHWAY_UP	-2.2	0.000	0.000
DACOSTA_UV_RESPONSE_VIA_ERCC3_DN	-2.19	0.000	0.000
PUJANA_BRCA2_PCC_NETWORK	-2.18	0.000	0.000
RIGGI_EWING_SARCOMA_PROGENITOR_DN	-2.18	0.000	0.000
GU_PDEF_TARGETS_UP	-2.18	0.000	0.000
ZHU_CMV_ALL_DN	-2.18	0.000	0.000
BENPORATH_PROLIFERATION	-2.18	0.000	0.000
KAN_RESPONSE_TO_ARSENIC_TRIOXIDE	-2.17	0.000	0.000
WANG_METHYLATED_IN_BREAST_CANCER	-2.17	0.000	0.000
KEGG_ECM_RECEPTOR_INTERACTION	-2.15	0.000	0.000
SHI_SPARC_TARGETS_UP	-2.15	0.000	0.000
KINSEY_TARGETS_OF_EWSR1_FLII_FUSION_UP	-2.15	0.000	0.000
KEGG_DNA_REPLICATION	-2.15	0.000	0.000
REACTOME_DNA_STRAND_ELONGATION	-2.14	0.000	0.000
CHANG_CYCLING_GENES	-2.13	0.000	0.000
ZHAN_MULTIPLE_MYELOMA_PR_UP	-2.13	0.000	0.000
REACTOME_CELL_CYCLE_MITOTIC	-2.12	0.000	0.000
KIM_WT1_TARGETS_12HR_DN	-2.12	0.000	0.000
MOLENAAR_TARGETS_OF_CCND1_AND_CDK4_DN	-2.12	0.000	0.000
SIMBULAN_UV_RESPONSE_IMMORTALIZED_DN	-2.12	0.000	0.000
ROZANOV_MMP14_TARGETS_SUBSET	-2.12	0.000	0.000
ONDER_CDH1_TARGETS_1_DN	-2.11	0.000	0.001
MANALO_HYPOXIA_DN	-2.11	0.000	0.001
REACTOME_ACTIVATION_OF_THE_PRE_REPLICATIVE_COMPLEX	-2.11	0.000	0.001
ZHU_CMV_24_HR_DN	-2.1	0.000	0.001
SENGUPTA_NASOPHARYNGEAL_CARCINOMA_WITH_LMP1_UP	-2.1	0.000	0.001
WAKASUGI_HAVE_ZNF143_BINDING_SITES	-2.1	0.000	0.001
GENTILE_UV_HIGH_DOSE_DN	-2.1	0.000	0.001
FINETTI_BREAST_CANCER_BASAL_VS_LUMINAL	-2.1	0.000	0.001
AMIT_EGF_RESPONSE_60_HELA	-2.08	0.000	0.001
VERRECCHIA_DELAYED_RESPONSE_TO_TGFB1	-2.08	0.000	0.001
KRIGE_AMINO_ACID_DEPRIVATION	-2.08	0.000	0.001
DE_YY1_TARGETS_DN	-2.08	0.000	0.001
MITSIADES_RESPONSE_TO_APLIDIN_DN	-2.08	0.000	0.001
LIEN_BREAST_CARCINOMA_METAPLASTIC_VS_DUCTAL_UP	-2.07	0.000	0.001
OSWALD_HEMATOPOIETIC_STEM_CELL_IN_COLLAGEN_GEL_UP	-2.07	0.000	0.001
OSWALD_HEMATOPOIETIC_STEM_CELL_IN_COLLAGEN_GEL_DN	-2.07	0.000	0.001
BIOCARTA_SPPA_PATHWAY	-2.06	0.000	0.002
HOFFMANN_LARGE_TO_SMALL_PRE_BII_LYMPHOCYTE_UP	-2.06	0.000	0.002
DASU_IL6_SIGNALING_SCAR_DN	-2.06	0.000	0.002
FURUKAWA_DUSP6_TARGETS_PCI35_DN	-2.06	0.000	0.002
LIAO_HAVE_SOX4_BINDING_SITES	-2.06	0.000	0.002
CHIARADONNA_NEOPLASTIC_TRANSFORMATION_KRAS_CDC25_UP	-2.05	0.000	0.001
SESTO_RESPONSE_TO_UV_C6	-2.05	0.000	0.002
MAHADEVAN_RESPONSE_TO_MP470_UP	-2.05	0.000	0.002
ONDER_CDH1_TARGETS_2_UP	-2.05	0.000	0.002
ZHAN_LATE_DIFFERENTIATION_GENES_UP	-2.05	0.000	0.002
SESTO_RESPONSE_TO_UV_C8	-2.05	0.000	0.002
KEGG_P53_SIGNALING_PATHWAY	-2.05	0.000	0.002
SAGIV_CD24_TARGETS_DN	-2.05	0.000	0.002
LEE_EARLY_T_LYMPHOCYTE_UP	-2.05	0.000	0.002
TURASHVILI_BREAST_LOBULAR_CARCINOMA_VS_LOBULAR_NORMAL_DN	-2.04	0.000	0.002

CD133- (continued)

Gene Set Name	NES	p-value	FDR
GHO_ATF5_TARGETS_DN	-2.04	0.000	0.002
GRAHAM_CML_QUIESCENT_VS_NORMAL_QUIESCENT_UP	-2.04	0.000	0.002
FRASOR_RESPONSE_TO_SERM_OR_FULVESTRANT_DN	-2.04	0.018	0.002
BIOCARTA_DREAM_PATHWAY	-2.03	0.000	0.002
AMIT_SERUM_RESPONSE_60_MCF10A	-2.03	0.000	0.002
GEORGES_TARGETS_OF_MIR192_AND_MIR215	-2.03	0.000	0.002
REACTOME_MITOTIC_PROMETAPHASE	-2.02	0.000	0.002
NAGASHIMA_NRG1_SIGNALING_UP	-2.02	0.000	0.002
SESTO_RESPONSE_TO_UV_C3	-2.02	0.000	0.002
SENESE_HDAC3_TARGETS_UP	-2.02	0.000	0.002
NAKAMURA_CANCER_MICROENVIRONMENT_DN	-2.02	0.000	0.002
HUANG_DASATINIB_RESISTANCE_UP	-2.02	0.000	0.002
KEGG_FOCAL_ADHESION	-2.02	0.000	0.002
VERRECCHIA_RESPONSE_TO_TGFB1_C4	-2.01	0.000	0.002
TRAYNOR_RETT_SYNDROM_UP	-2.01	0.000	0.002
WU_HBX_TARGETS_1_UP	-2.01	0.000	0.002
MORI_IMMATURE_B_LYMPHOCYTE_DN	-2.01	0.000	0.002
MAHADEVAN_RESPONSE_TO_MP470_DN	-2.01	0.000	0.003
DACOSTA_UV_RESPONSE_VIA_ERCC3_COMMON_DN	-2	0.000	0.003
AMIT_EGF_RESPONSE_40_MCF10A	-2	0.000	0.003
NAKAYAMA_SOFT_TISSUE_TUMORS_PCA2_UP	-2	0.000	0.003
LEE_LIVER_CANCER_HEPATOBLAST	-2	0.000	0.003
FINETTI_BREAST_CANCER_KINOME_RED	-1.99	0.000	0.003
CROONQUIST_STROMAL_STIMULATION_UP	-1.99	0.000	0.003
ZHANG_PROLIFERATING_VS_QUIESCENT	-1.99	0.000	0.003
BIDUS_METASTASIS_UP	-1.99	0.000	0.003
TURASHVILI_BREAST_LOBULAR_CARCINOMA_VS_DUCTAL_NORMAL_UP	-1.99	0.000	0.003
BACOLOD_RESISTANCE_TO_ALKYLATING_AGENTS_UP	-1.99	0.000	0.003
HORIUCHI_WTAP_TARGETS_DN	-1.99	0.000	0.003
PYEON_CANCER_HEAD_AND_NECK_VS_CERVICAL_UP	-1.99	0.000	0.003
HINATA_NFKB_TARGETS_KERATINOCYTE_UP	-1.99	0.000	0.003
AMIT_EGF_RESPONSE_60_MCF10A	-1.98	0.000	0.003
SENESE_HDAC1_TARGETS_UP	-1.98	0.000	0.004
WATTEL_AUTONOMOUS_THYROID_ADENOMA_DN	-1.98	0.000	0.004
PYEON_HPV_POSITIVE_TUMORS_UP	-1.98	0.000	0.004
LINDGREN_BLADDER_CANCER_CLUSTER_1_DN	-1.97	0.000	0.004
AMIT_SERUM_RESPONSE_40_MCF10A	-1.97	0.000	0.004
KARAKAS_TGFB1_SIGNALING	-1.97	0.000	0.004
TAVAZOIE_METASTASIS	-1.97	0.000	0.004
ODONNELL_TARGETS_OF_MYC_AND_TFRC_DN	-1.97	0.000	0.004
REACTOME_MITOTIC_M_M_G1_PHASES	-1.97	0.000	0.004
WEST_ADRENOCORTICAL_TUMOR_UP	-1.96	0.000	0.004
FRIDMAN_SENESCENCE_UP	-1.96	0.000	0.004
AMUNDSON_GAMMA_RADIATION_RESPONSE	-1.96	0.000	0.004
WAMUNYOKOLI_OVARIAN_CANCER_LMP_DN	-1.96	0.000	0.004
GERY_CEBP_TARGETS	-1.96	0.000	0.004
NEWMAN_ERCC6_TARGETS_UP	-1.95	0.000	0.004
KAUFFMANN_MELANOMA_RELAPSE_UP	-1.95	0.000	0.004
WEI_MYCN_TARGETS_WITH_E_BOX	-1.95	0.000	0.004
VECCHI_GASTRIC_CANCER_ADVANCED_VS_EARLY_UP	-1.95	0.000	0.004
REACTOME_E2F_TRANSCRIPTIONAL_TARGETS_AT_G1_S	-1.95	0.000	0.005
EGUCHI_CELL_CYCLE_RB1_TARGETS	-1.94	0.000	0.005
LY_AGING_OLD_DN	-1.94	0.000	0.005
LEE_LIVER_CANCER_DENA_UP	-1.94	0.000	0.005
REACTOME_G1_S_TRANSITION	-1.94	0.000	0.005
LY_AGING_MIDDLE_DN	-1.94	0.000	0.005
REACTOME_ACTIVATION_OF_ATR_IN_RESPONSE_TO_REPLICATION_STRESS	-1.94	0.000	0.005
NAGASHIMA_EGF_SIGNALING_UP	-1.93	0.000	0.005
GENTILE_RESPONSE_CLUSTER_D3	-1.93	0.000	0.005
IGARASHI_ATF4_TARGETS_DN	-1.93	0.000	0.005
KAUFFMANN_DNA_REPAIR_GENES	-1.93	0.000	0.005
WANG_SMARCE1_TARGETS_UP	-1.93	0.000	0.005
FARMER_BREAST_CANCER_CLUSTER_2	-1.93	0.000	0.006
RHEIN_ALL_GLUCOCORTICOID_THERAPY_DN	-1.93	0.000	0.006
BENPORATH_CYCLING_GENES	-1.93	0.000	0.006
BEGUM_TARGETS_OF_PAX3_FOXO1_FUSION_UP	-1.92	0.000	0.006
GRAHAM_CML_QUIESCENT_VS_NORMAL_DIVIDING_UP	-1.92	0.000	0.006
RHODES_UNDIFFERENTIATED_CANCER	-1.92	0.000	0.006
GENTILE_UV_RESPONSE_CLUSTER_D2	-1.92	0.000	0.006
UZONYI_RESPONSE_TO_LEUKOTRIENE_AND_THROMBIN	-1.92	0.000	0.006
CAIRO_HEPATOBLASTOMA_CLASSES_UP	-1.92	0.000	0.006
WIKMAN_ASBESTOS_LUNG_CANCER_DN	-1.92	0.000	0.006
TARTE_PLASMA_CELL_VS_PLASMABLAST_DN	-1.92	0.000	0.006
LIU_VMYB_TARGETS_UP	-1.92	0.000	0.006
HINATA_NFKB_TARGETS_FIBROBLAST_UP	-1.91	0.000	0.006
ZHANG_RESPONSE_TO_IKK_INHIBITOR_AND_TNF_DN	-1.91	0.019	0.006
LI_WILMS_TUMOR_VS_FETAL_KIDNEY_1_DN	-1.91	0.000	0.007
CERVERA_SDHB_TARGETS_1_UP	-1.91	0.000	0.007
PUJANA_CHEK2_PCC_NETWORK	-1.91	0.000	0.007
BIOCARTA_CDMAC_PATHWAY	-1.91	0.019	0.007
KONDO_EZH2_TARGETS	-1.91	0.000	0.007
REACTOME_G2_M_CHECKPOINTS	-1.9	0.000	0.008
JEON_SMAD6_TARGETS_UP	-1.9	0.000	0.008
KEGG_CELL_CYCLE	-1.9	0.000	0.008
NUYTTEN_EZH2_TARGETS_UP	-1.9	0.000	0.008
SENESE_HDAC1_AND_HDAC2_TARGETS_UP	-1.9	0.000	0.008
WOO_LIVER_CANCER_RECURRENCE_UP	-1.89	0.000	0.008
PUJANA_BRCA_CENTERED_NETWORK	-1.89	0.000	0.008
STEIN_ESR1_TARGETS	-1.89	0.000	0.008
LINDGREN_BLADDER_CANCER_CLUSTER_2A_DN	-1.89	0.000	0.009
REACTOME_CELL_CYCLE_CHECKPOINTS	-1.89	0.000	0.009
CHEN_LVAD_SUPPORT_OF_FAILING_HEART_UP	-1.89	0.000	0.009
REACTOME_PD1_SIGNALING	-1.89	0.000	0.009
KEGG_BLADDER_CANCER	-1.89	0.000	0.009
HALMOS_CEBPA_TARGETS_UP	-1.89	0.000	0.009
VECCHI_GASTRIC_CANCER_EARLY_UP	-1.89	0.000	0.009
BHATI_G2M_ARREST_BY_2METHOXYESTRADIOL_UP	-1.88	0.000	0.009
BERENJENO_TRANSFORMED_BY_RHOA_REVERSIBLY_DN	-1.88	0.000	0.009

CD133- (continued)

Gene Set Name	NES	p-value	FDR
TANG_SENESCENCE_TP53_TARGETS_DN	-1.88	0.000	0.009
REACTOME_PHOSPHORYLATION_OF_CD3_AND_TCR_ZETA_CHAINS	-1.88	0.000	0.009
REACTOME_INTEGRIN_CELL_SURFACE_INTERACTIONS	-1.88	0.000	0.009
REACTOME_SYNTHESIS_OF_DNA	-1.87	0.000	0.010
DAVICIONI_TARGETS_OF_PAX_FOXO1_FUSIONS_UP	-1.87	0.000	0.010
BILD_E2F3_ONCOGENIC_SIGNATURE	-1.87	0.000	0.010
LINDGREN_BLADDER_CANCER_CLUSTER_3_UP	-1.87	0.000	0.010
CASORELLI_ACUTE_PROMYELOCYTIC_LEUKEMIA_DN	-1.87	0.000	0.010
BENPORATH_ES_CORE_NINE_CORRELATED	-1.87	0.000	0.010
BROWNE_HCMV_INFECTION_2HR_DN	-1.87	0.000	0.010
ELVIDGE_HYPOXIA_UP	-1.86	0.000	0.011
CHIANG_LIVER_CANCER_SUBCLASS_PROLIFERATION_UP	-1.86	0.000	0.011
LINDGREN_BLADDER_CANCER_CLUSTER_2B	-1.86	0.000	0.011
XU_RESPONSE_TO_TRETINOIN_AND_NSC682994_UP	-1.86	0.000	0.011
KEGG_LEISHMANIA_INFECTION	-1.86	0.000	0.011
GAUSSMANN_MLL_AF4_FUSION_TARGETS_F_DN	-1.86	0.000	0.011
POOLA_INVASIVE_BREAST_CANCER_UP	-1.86	0.000	0.011
ELVIDGE_HYPOXIA_BY_DMOG_UP	-1.86	0.000	0.011
REACTOME_MAPK_TARGETS_NUCLEAR_EVENTS_MEDIATED_BY_MAP_KINASES	-1.85	0.000	0.011
REACTOME_LAGGING_STRAND_SYNTHESIS	-1.85	0.000	0.012
BOYAULT_LIVER_CANCER_SUBCLASS_G123_UP	-1.85	0.000	0.012
BIOCARTA_LYM_PATHWAY	-1.85	0.000	0.012
GEORGES_CELL_CYCLE_MIR192_TARGETS	-1.85	0.000	0.012
ENK_UV_RESPONSE_KERATINOCYTE_DN	-1.85	0.000	0.012
REACTOME_BASIGIN_INTERACTIONS	-1.84	0.000	0.013
PRAMOONJAGO_SOX4_TARGETS_UP	-1.84	0.000	0.013
BROWNE_HCMV_INFECTION_10HR_DN	-1.84	0.000	0.014
TSAI_RESPONSE_TO_RADIATION_THERAPY	-1.84	0.000	0.014
CAFFAREL_RESPONSE_TO_THC_24HR_5_DN	-1.84	0.000	0.014
PARK_APL_PATHOGENESIS_UP	-1.84	0.000	0.014
WU_APOPTOSIS_BY_CDKN1A_VIA_TP53	-1.84	0.000	0.014
SCHAVOLT_TARGETS_OF_TP53_AND_TP63	-1.84	0.000	0.014
BOYAULT_LIVER_CANCER_SUBCLASS_G23_UP	-1.83	0.000	0.014
JI_RESPONSE_TO_FSH_DN	-1.83	0.000	0.014
REACTOME_S_PHASE	-1.83	0.000	0.015
TAKEDA_TARGETS_OF_NUP98_HOXA9_FUSION_6HR_UP	-1.83	0.000	0.015
INGA_TP53_TARGETS	-1.83	0.018	0.015
LAIHO_COLORECTAL_CANCER_SERRATED_UP	-1.83	0.000	0.015
GOLDRATH_ANTIGEN_RESPONSE	-1.82	0.000	0.015
VANTVEER_BREAST_CANCER_METASTASIS_DN	-1.82	0.000	0.015
KUMAMOTO_RESPONSE_TO_NUTLIN_3A_DN	-1.82	0.000	0.016
BUYTAERT_PHOTODYNAMIC_THERAPY_STRESS_DN	-1.82	0.000	0.016
KEGG_HOMOLOGOUS_RECOMBINATION	-1.82	0.000	0.016
KEGG_DILATED_CARDIOMYOPATHY	-1.82	0.000	0.016
HELLER_SILENCED_BY_METHYLATION_DN	-1.82	0.000	0.016
CHARAFE_BREAST_CANCER_BASAL_VS_MESENCHYMAL_DN	-1.82	0.019	0.016
REACTOME_SIGNALING_BY_TGF_BETA	-1.82	0.000	0.017
LU_TUMOR_ENDOTHELIAL_MARKERS_UP	-1.82	0.000	0.017
BENPORATH_NOS_TARGETS	-1.82	0.000	0.017
WU_HBX_TARGETS_2_DN	-1.81	0.019	0.017
GRAHAM_CML_QUIESCENT_VS_CML_DIVIDING_UP	-1.81	0.041	0.017
KALMA_E2F1_TARGETS	-1.81	0.000	0.017
BIOCARTA_AMI_PATHWAY	-1.81	0.000	0.017
MMS_MOUSE_LYMPH_HIGH_4HRS_UP	-1.81	0.000	0.017
KEGG_NUCLEOTIDE_EXCISION_REPAIR	-1.81	0.000	0.018
VANHARANTA_UTERINE_FIBROID_UP	-1.81	0.000	0.018
NUYTTEN_EZH2_TARGETS_DN	-1.81	0.000	0.018
FONTAINE_THYROID_TUMOR_UNCERTAIN_MALIGNANCY_DN	-1.8	0.016	0.018
KEGG_MISMATCH_REPAIR	-1.8	0.000	0.018
OSADA_ASCL1_TARGETS_DN	-1.8	0.000	0.018
REACTOME_POLYMERASE_SWITCHING	-1.8	0.000	0.018
LIANG_SILENCED_BY_METHYLATION_DN	-1.8	0.023	0.018
BILD_HRAS_ONCOGENIC_SIGNATURE	-1.8	0.000	0.018
SMIRNOV_CIRCULATING_ENDOTHELIOCYTES_IN_CANCER_UP	-1.8	0.000	0.018
LEE_LIVER_CANCER_SURVIVAL_DN	-1.8	0.000	0.019
AMIT_DELAYED_EARLY_GENES	-1.8	0.000	0.019
REACTOME_EXTENSION_OF_TELOMERES	-1.8	0.022	0.019
PICCALUGA_ANGIOIMMUNOBLASTIC_LYMPHOMA_UP	-1.8	0.000	0.019
GAURNIER_PSMD4_TARGETS	-1.8	0.000	0.019
CHOW_RASSF1_TARGETS_UP	-1.8	0.000	0.019
MATTIOLI_MGUS_VS_MULTIPLE_MYELOMA	-1.79	0.000	0.019
RODRIGUES_THYROID_CARCINOMA_DN	-1.79	0.000	0.019
MAHADEVAN_IMATINIB_RESISTANCE_UP	-1.79	0.000	0.019
OUYANG_PROSTATE_CANCER_PROGRESSION_UP	-1.79	0.000	0.019
KANG_IMMORTALIZED_BY_TERT_UP	-1.79	0.000	0.019
GINESTIER_BREAST_CANCER_ZNF217_AMPLIFIED_UP	-1.79	0.000	0.019
IZADPANAH_STEM_CELL_ADIPOSE_VS_BONE_UP	-1.79	0.000	0.020
MURATA_VIRULENCE_OF_H_PILORI	-1.79	0.044	0.020
BIOCARTA_MCM_PATHWAY	-1.79	0.000	0.020
AMIT_SERUM_RESPONSE_240_MCF10A	-1.79	0.000	0.020
LEE_NEURAL_CREST_STEM_CELL_UP	-1.79	0.000	0.020
RODRIGUES_NTN1_TARGETS_UP	-1.78	0.000	0.020
FERRANDO_HOX11_NEIGHBORS	-1.78	0.000	0.020
FULCHER_INFLAMMATORY_RESPONSE_LECTIN_VS_LPS_UP	-1.78	0.000	0.021
OSMAN_BLADDER_CANCER_UP	-1.78	0.000	0.021
HOSHIDA_LIVER_CANCER_SUBCLASS_S1	-1.78	0.000	0.021
LIEN_BREAST_CARCINOMA_METAPLASTIC	-1.78	0.000	0.021
KEGG_TGF_BETA_SIGNALING_PATHWAY	-1.78	0.000	0.021
CHEMNITZ_RESPONSE_TO_PROSTAGLANDIN_E2_UP	-1.78	0.000	0.021
LU_TUMOR_VASCULATURE_UP	-1.78	0.000	0.022
VALK_AML_CLUSTER_6	-1.78	0.000	0.022
KENNY_CTNNB1_TARGETS_UP	-1.78	0.000	0.022
GAUSSMANN_MLL_AF4_FUSION_TARGETS_E_UP	-1.77	0.000	0.022
RIZ_ERYTHROID_DIFFERENTIATION_HEMGN	-1.77	0.000	0.022
DEBIASI_APOPTOSIS_BY_REOVIRUS_INFECTION_UP	-1.77	0.000	0.022
BIOCARTA_GCR_PATHWAY	-1.77	0.000	0.022
GRAHAM_NORMAL_QUIESCENT_VS_NORMAL_DIVIDING_UP	-1.77	0.000	0.022

CD133- (continued)

Gene Set Name	NES	p-value	FDR
REACTOME_DNA_REPLICATION_PRE_INITIATION	-1.77	0.000	0.022
BIOCARTA_NGF_PATHWAY	-1.77	0.000	0.022
KOBAYASHI_EGFR_SIGNALING_6HR_DN	-1.77	0.000	0.023
AMIT_EGF_RESPONSE_120_HELA	-1.77	0.000	0.023
GENTILE_UV_RESPONSE_CLUSTER_D1	-1.77	0.019	0.024
REACTOME_GLOBAL_GENOMIC_NER	-1.77	0.000	0.024
WEST_ADRENOCORTICAL_CARCINOMA_VS_ADENOMA_DN	-1.76	0.000	0.024
KIM_WT1_TARGETS_UP	-1.76	0.000	0.024
HEIDENBLAD_AMPLIFIED_IN_PANCREATIC_CANCER	-1.76	0.000	0.024
BOYLAN_MULTIPLE_MYELOMA_C_D_UP	-1.76	0.000	0.024
WILCOX_PRESPONSE_TO_ROGESTERONE_UP	-1.76	0.000	0.025
LUI_THYROID_CANCER_CLUSTER_4	-1.76	0.000	0.025
MARZEC_IL2_SIGNALING_UP	-1.76	0.000	0.025
MORI_MATURE_B_LYMPHOCYTE_DN	-1.76	0.000	0.025
WU_CELL_MIGRATION	-1.76	0.000	0.026
WNT_SIGNALING	-1.75	0.000	0.026
LIU_PROSTATE_CANCER_DN	-1.75	0.000	0.027
BIOCARTA_ATM_PATHWAY	-1.75	0.018	0.027
CHEN_HOXA5_TARGETS_9HR_UP	-1.75	0.000	0.027
BIOCARTA_IL4_PATHWAY	-1.75	0.022	0.027
NGUYEN_NOTCH1_TARGETS_DN	-1.75	0.000	0.027
KAUFFMANN_DNA_REPLICATION_GENES	-1.75	0.000	0.027
KRIGE_RESPONSE_TO_TOSEDOSTAT_6HR_UP	-1.75	0.000	0.027
DAZARD_RESPONSE_TO_UV_NHEK_DN	-1.75	0.000	0.027
BORCZUK_MALIGNANT_MESOTHELIOMA_UP	-1.75	0.000	0.027
BROWNE_HCMV_INFECTION_30MIN_UP	-1.75	0.000	0.027
ADDYA_ERYTHROID_DIFFERENTIATION_BY_HEMIN	-1.75	0.000	0.027
FOSTER_INFLAMMATORY_RESPONSE_LPS_DN	-1.75	0.000	0.027
NOJIMA_SFRP2_TARGETS_UP	-1.75	0.000	0.027
BIOCARTA_PLCE_PATHWAY	-1.74	0.020	0.027
ROY_WOUND_BLOOD_VESSEL_UP	-1.74	0.000	0.027
CROMER_TUMORIGENESIS_UP	-1.74	0.000	0.028
OLSSON_E2F3_TARGETS_DN	-1.74	0.000	0.028
DACOSTA_UV_RESPONSE_VIA_ERCC3_TTD_DN	-1.74	0.000	0.028
SCHUHMACHER_MYC_TARGETS_UP	-1.74	0.000	0.028
WANG_SMARCE1_TARGETS_DN	-1.74	0.000	0.028
REACTOME_CYTOSOLIC_TRNA_AMINOACYLATION	-1.74	0.000	0.028
COATES_MACROPHAGE_M1_VS_M2_UP	-1.74	0.000	0.028
NAGASHIMA_NRG1_SIGNALING_DN	-1.74	0.000	0.029
REACTOME_MAP_KINASES_ACTIVATION_IN_TLR_CASCADE	-1.74	0.016	0.029
IWANAGA_CARCINOGENESIS_BY_KRAS_DN	-1.73	0.000	0.029
REACTOME_SIGNALING_BY_PDGF	-1.73	0.000	0.029
CROMER_METASTASIS_DN	-1.73	0.000	0.029
BERTUCCI_INVASIVE_CARCINOMA_DUCTAL_VS_LOBULAR_DN	-1.73	0.000	0.029
MUELLER_COMMON_TARGETS_OF_AML_FUSIONS_DN	-1.73	0.017	0.029
BERENJENO_ROCK_SIGNALING_NOT_VIA_RHOA_DN	-1.73	0.000	0.030
NIKOLSKY_BREAST_CANCER_1Q32_AMPLICON	-1.73	0.020	0.030
PICCALUGA_ANGIOIMMUNOBLASTIC_LYMPHOMA_DN	-1.73	0.000	0.030
BIOCARTA_GPCR_PATHWAY	-1.72	0.000	0.031
MOHANKUMAR_TLX1_TARGETS_DN	-1.72	0.000	0.031
VERRECCHIA_RESPONSE_TO_TGFB1_C5	-1.72	0.019	0.031
SASAKI_ADULT_T_CELL_LEUKEMIA	-1.72	0.000	0.031
NUYTTEN_NIPP1_TARGETS_UP	-1.72	0.000	0.031
SEKI_INFLAMMATORY_RESPONSE_LPS_UP	-1.72	0.000	0.031
DAZARD_RESPONSE_TO_UV_SCC_DN	-1.72	0.000	0.031
BROWNE_HCMV_INFECTION_14HR_DN	-1.72	0.000	0.032
BIOCARTA_INTEGRIN_PATHWAY	-1.72	0.020	0.032
MORI_EMU_MYC_LYMPHOMA_BY_ONSET_TIME_UP	-1.72	0.000	0.032
ZHANG_BREAST_CANCER_PROGENITORS_UP	-1.72	0.000	0.032
CERVERA_SDHB_TARGETS_2	-1.72	0.000	0.033
BIOCARTA_LAIR_PATHWAY	-1.72	0.019	0.033
MANALO_HYPOXIA_UP	-1.72	0.000	0.033
TOYOTA_TARGETS_OF_MIR34B_AND_MIR34C	-1.72	0.000	0.033
CHEN_HOXA5_TARGETS_6HR_UP	-1.71	0.000	0.033
SMID_BREAST_CANCER_BASAL_UP	-1.71	0.000	0.033
SEITZ_NEOPLASTIC_TRANSFORMATION_BY_8P_DELETION_UP	-1.71	0.000	0.034
GAJATE_RESPONSE_TO_TRABECTEDIN_UP	-1.71	0.019	0.034
ST_INTEGRIN_SIGNALING_PATHWAY	-1.71	0.000	0.034
SARRIO_EPITHELIAL_MESENCHYMAL_TRANSITION_UP	-1.71	0.000	0.034
KEGG_NOD_LIKE_RECEPTOR_SIGNALING_PATHWAY	-1.71	0.000	0.034
ITO_PTTG1_TARGETS_UP	-1.71	0.036	0.034
LY_AGING_MIDDLE_UP	-1.71	0.000	0.034
REACTOME_E2F_MEDIATED_REGULATION_OF_DNA_REPLICATION	-1.71	0.000	0.035
TENEDINI_MEGAKARYOCYTE_MARKERS	-1.71	0.000	0.035
AMIT_EGF_RESPONSE_20_MCF10A	-1.7	0.020	0.035
BIOCARTA_VITCB_PATHWAY	-1.7	0.033	0.035
SPIRA_SMOKERS_LUNG_CANCER_UP	-1.7	0.000	0.035
MCCLUNG_DELTA_FOSB_TARGETS_2WK	-1.7	0.000	0.035
LY_AGING_PREMATURE_DN	-1.7	0.000	0.035
LANDIS_BREAST_CANCER_PROGRESSION_DN	-1.7	0.000	0.035
KHETCHOUMIAN_TRIM24_TARGETS_UP	-1.7	0.020	0.035
REACTOME_TRANSLOCATION_OF_ZAP70_TO_IMMUNOLOGICAL_SYNAPSE	-1.7	0.000	0.035
BIOCARTA_CFTR_PATHWAY	-1.7	0.000	0.035
DASU_IL6_SIGNALING_SCAR_UP	-1.7	0.000	0.036
LOPEZ_MESOTHELIOMA_SURVIVAL_WORST_VS_BEST_UP	-1.7	0.019	0.036
REACTOME_NUCLEOTIDE_EXCISION_REPAIR	-1.7	0.032	0.036
SEITZ_NEOPLASTIC_TRANSFORMATION_BY_8P_DELETION_DN	-1.7	0.000	0.036
FOURNIER_ACINAR_DEVELOPMENT_LATE_DN	-1.69	0.000	0.037
RHODES_CANCER_META_SIGNATURE	-1.69	0.026	0.037
BIOCARTA_IL17_PATHWAY	-1.69	0.022	0.037
MCMURRAY_TP53_HRAS_COOPERATION_RESPONSE_UP	-1.69	0.018	0.038
KEGG_HYPERTROPHIC_CARDIOMYOPATHY_HCM	-1.69	0.000	0.039
PUJANA_BREAST_CANCER_LIT_INT_NETWORK	-1.68	0.000	0.040
MODY_HIPPOCAMPUS_NEONATAL	-1.68	0.019	0.040
WAMUNYOKOLI_OVARIAN_CANCER_GRADES_1_2_DN	-1.68	0.018	0.040
FRIDMAN_IMMORTALIZATION_DN	-1.68	0.018	0.040
NAKAYAMA_SOFT_TISSUE_TUMORS_PCA1_UP	-1.68	0.000	0.040
SEIDEN_MET_SIGNALING	-1.68	0.036	0.040

CD133- (continued)			
**Gene Set Name**	**NES**	**p-value**	**FDR**
MARKEY_RB1_CHRONIC_LOF_UP	-1.68	0.000	0.040
MARTINEZ_RESPONSE_TO_TRABECTEDIN_DN	-1.68	0.000	0.041
CHUANG_OXIDATIVE_STRESS_RESPONSE_UP	-1.68	0.000	0.041
MORI_LARGE_PRE_BII_LYMPHOCYTE_UP	-1.68	0.000	0.041
BILBAN_B_CLL_LPL_DN	-1.68	0.018	0.042
REACTOME_DNA_REPAIR	-1.68	0.000	0.042
KYNG_DNA_DAMAGE_BY_4NQO_OR_UV	-1.68	0.019	0.042
TURASHVILI_BREAST_DUCTAL_CARCINOMA_VS_LOBULAR_NORMAL_UP	-1.67	0.000	0.042
BROWNE_HCMV_INFECTION_18HR_DN	-1.67	0.000	0.042
KEGG_INTESTINAL_IMMUNE_NETWORK_FOR_IGA_PRODUCTION	-1.67	0.018	0.043
DAUER_STAT3_TARGETS_UP	-1.67	0.000	0.043
BIOCARTA_IL2_PATHWAY	-1.67	0.040	0.043
KEGG_PRION_DISEASES	-1.67	0.038	0.043
YU_MYC_TARGETS_UP	-1.67	0.000	0.043
XU_HGF_TARGETS_INDUCED_BY_AKT1_6HR	-1.67	0.000	0.043
RAMALHO_STEMNESS_UP	-1.67	0.000	0.043
ONDER_CDH1_TARGETS_3_UP	-1.67	0.000	0.043
SMID_BREAST_CANCER_RELAPSE_IN_BONE_DN	-1.67	0.000	0.043
RASHI_RESPONSE_TO_IONIZING_RADIATION_2	-1.67	0.000	0.043
KRIGE_RESPONSE_TO_TOSEDOSTAT_24HR_UP	-1.66	0.000	0.045
DASU_IL6_SIGNALING_UP	-1.66	0.032	0.046
PUJANA_BRCA1_PCC_NETWORK	-1.66	0.000	0.046
GARY_CD5_TARGETS_DN	-1.66	0.000	0.046
HOOI_ST7_TARGETS_UP	-1.66	0.000	0.046
HUANG_FOXA2_TARGETS_UP	-1.66	0.000	0.047
SUNG_METASTASIS_STROMA_DN	-1.66	0.000	0.047
MCBRYAN_PUBERTAL_BREAST_5_6WK_UP	-1.66	0.000	0.048
TAKADA_GASTRIC_CANCER_COPY_NUMBER_DN	-1.66	0.000	0.048
BIOCARTA_BAD_PATHWAY	-1.66	0.000	0.048
DAZARD_UV_RESPONSE_CLUSTER_G6	-1.66	0.000	0.048
SENESE_HDAC2_TARGETS_UP	-1.66	0.000	0.048
GARGALOVIC_RESPONSE_TO_OXIDIZED_PHOSPHOLIPIDS_TURQUOISE_DN	-1.65	0.000	0.048
BIOCARTA_PLATELETAPP_PATHWAY	-1.65	0.000	0.048
GRUETZMANN_PANCREATIC_CANCER_UP	-1.65	0.000	0.048
SANA_TNF_SIGNALING_DN	-1.65	0.000	0.048
CHUANG_OXIDATIVE_STRESS_RESPONSE_DN	-1.65	0.087	0.049
FARMER_BREAST_CANCER_BASAL_VS_LULMINAL	-1.65	0.000	0.049
BERTUCCI_MEDULLARY_VS_DUCTAL_BREAST_CANCER_DN	-1.65	0.000	0.049
BIOCARTA_NTHI_PATHWAY	-1.65	0.019	0.049
