# Supplementary figures and images for: Iterative sorting reveals CD133+ and CD133- melanoma cells as phenotypically distinct populations
Source: BMC Cancer. 2016 Sep 9;16(1):726. doi: 10.1186/s12885-016-2759-2 (PMC5017126; doi:10.1186/s12885-016-2759-2)

LM-MEL-15

LM-MEL-34

LM-MEL-62

CD133+

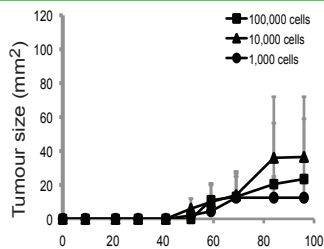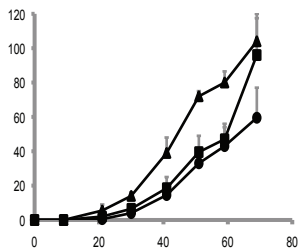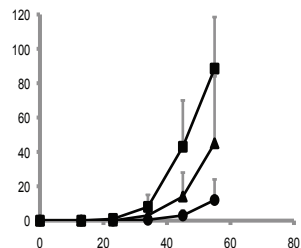

CD133-

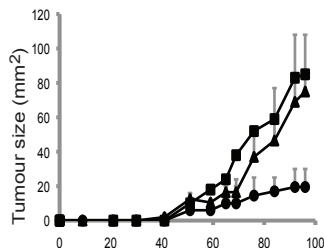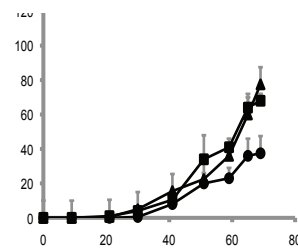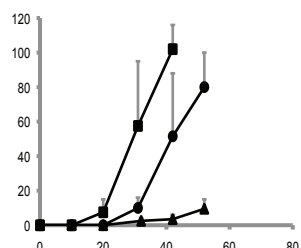

Days

Supplement: Additional file 2: Figure S1. — CD133+ and CD133- cells have similar frequency of tumour-initiating cells. Serial dilution of CD133+ and CD133- cell used in sub-cutaneous xenograft. Square, 105 cells; triangle, 104 cells; circle, 103 cells. Average (+/- SD) tumour volume measured over time, 3–5 mice/group. Data representative of 2 independent replicate experiments (PDF 203 kb) [file 12885_2016_2759_MOESM2_ESM.pdf]
